# Results of longitudinal Nutri-D study: factors influencing winter and summer vitamin D status in a Caucasian population

**DOI:** 10.3389/fnut.2023.1253341

**Published:** 2023-11-15

**Authors:** Maša Hribar, Igor Pravst, Tina Pogačnik, Katja Žmitek

**Affiliations:** ^1^Nutrition Institute, Ljubljana, Slovenia; ^2^Biotechnical Faculty, University of Ljubljana, Ljubljana, Slovenia; ^3^VIST – Faculty of Applied Sciences, Ljubljana, Slovenia

**Keywords:** 25(OH)D, deficiency, sun exposure, individual typology angle, melanin index, Caucasian, Slovenia

## Abstract

**Introduction:**

Inadequate vitamin D status is a worldwide public health issue. In humans, vitamin D status is affected by diet, and even more by exposure to ultraviolet B (UVB) light and consequential endogenous synthesis. Various personal and environmental factors influence endogenous synthesis. Factors affecting vitamin D status were investigated in a prospective longitudinal cohort study with a summer and winter observation period.

**Methods:**

The final sample included 292 adults, of those 111 (38%) males and 181 (62%) females, with a mean age of 38.2 (±11.8) years from Slovenia who were not supplementing vitamin D. Serum 25-hydroxyvitamin D [25(OH)D] concentrations were measured in both periods; vitamin D intake, self-reported body mass index (BMI), and protective behaviors against sun were also recorded. Other measured parameters included measurements of constitutive skin color using the objective individual typology angle (ITA), and difference in the melanin index (ΔMI) for assessment of objective sun exposure.

**Results:**

In winter a high prevalence (63.4%) of insufficient vitamin D status (< 50 nmoL/L) was observed with higher odds ratios (OR) for insufficiency in those with a higher BMI and light ITA. During summer, insufficiency prevalence was low (5.5%), but half of the participants (50.0%) had suboptimal 25(OH)D concentration (< 75 nmol/L). In summer OR for suboptimal status were higher in those with obesity, lower ΔMI, light ITA, low vitamin D intake, and protective clothing behaviors.

**Conclusion:**

Using a series of measures, we showed that vitamin D status is hugely affected by several personal factors such as BMI, ITA, vitamin D intake, ΔMI, and protective behavior against the sun. This conclusion questions the usefulness of generalized population-level recommendations since personal factors are a major predictor of vitamin D status.

## 1. Introduction

Epidemiological studies in various populations have raised public health concerns because of the high prevalence of vitamin D (VitD) insufficiency and deficiency (1–9). Since it plays an important role in human physiology, low serum VitD concentrations may pose a health risk (2, 10). Vitamin D is a fat-soluble vitamin involved in calcium and phosphorus homeostasis and is therefore critical for bone health (11). Poor VitD status leads to rickets in children and osteomalacia in adults. Other studies have linked VitD deficiency to immune system function, non-skeletal health problems, disease development, and lower life expectancy, although causal evidence from randomized controlled trials is still limited (12, 13).

Sources of VitD for the human body are dietary intake and skin exposure to ultraviolet B (UVB) radiation with wavelengths of 290–315 nm. Exposure to sunlight is thought to account for up to 90% of the body’s VitD supply, but during the winter months sun exposure in most European countries does not result in the formation of VitD due to the low intensity of UVB radiation (14–16). The amount of VitD formed in the skin by UVB radiation depends on various environmental and personal factors, such as latitude, season, time of day, skin pigmentation, age, or use of sunscreen (17–23). It has already been established that constitutive skin pigmentation and/or skin phototype is an important predictor of VitD status when comparing people from different ethnic backgrounds (24–29), but how different levels of constitutive pigmentation affect VitD status in Caucasians has not yet been well described. To date, there has been only one study focusing on Caucasians, and its results contrast with studies of different ethnic backgrounds, where darker skin types seem to be more prone to deficiency (30).

Vitamin D is formed after UVB exposure to the skin; it enters the bloodstream in a complex with a D-binding protein and is later hydroxylated in the liver to 25-hydroxyvitamin D [25(OH)D], the most common biomarker for assessing VitD status. This form is later hydroxylated in the kidneys to 1, 25 hydroxyvitamin D, which is the active form of VitD (31). The excess VitD produced is stored in adipose tissue and/or partially degraded (32). Melanin pigment, which is crucial for skin color, may interfere with VitD photoproduction in the skin because it acts as a natural filter of UVB radiation in the epidermis, and thus competes for UVB photons with 7-dehydrocholesterol (7-DHC), a substrate for VitD synthesis (32).

Although there is no consensus on the recommended serum concentration of 25(OH)D, the definition of VitD deficiency generally includes serum concentrations of 25(OH)D that fall below 30 nmol/L (10–12 ng/mL) (33–36); concentrations below 50 nmol/L (20 ng/mL) are considered insufficient (33, 35–37), whereas concentrations above 75 nmol/L (30 ng/mL) are considered optimal by the Endocrine Society (34), In Slovenia (latitude 45° to 46° north) and in countries with similar or higher latitudes, UVB radiation in winter (November–April) is insufficient to stimulate VitD synthesis (3). In Slovenia a high prevalence of low VitD status with remarkable seasonal variations has been reported (1), highlighting the need for effective public health interventions.

In foods, VitD is mainly found in the form of ergocalciferol (D2) and cholecalciferol (D3) (38) and their derivatives. Foods may be a natural source of VitD or may be enriched with this vitamin. There are few natural dietary sources of VitD (e.g., fatty fish, fish liver oil, and sun-dried mushrooms), and they are not commonly consumed (5, 7). As a result, most VitD intake is achieved by foods that contain rather small amounts of VitD but are consumed more frequently, such as eggs and cheese (7, 39, 40). On the other hand, food fortification and bioaddition can be important sources of VitD, which may be voluntary or mandatory (41). The term fortification usually describes the process of adding D2 or D3 before or at the end of food processing (42), whereas the term bioaddition describes the process of increasing naturally occurring VitD during food production by feeding animals with VitD-enriched feed (for meat and eggs) or by irradiating fungi or yeast with UVB (42). Other sources of this vitamin include VitD-containing prescription medicines and food supplements.

Dietary intake of VitD is often far below recommended levels – both globally and in Slovenia (40, 43, 44). The daily reference value (DRV) for VitD intake (without endogenous synthesis) for the adult population is 15 μg/day as recommended by the European Food Safety Authority (EFSA), and 20 μg recommended by the German, Austrian, and Swiss Nutrition Association (D-A-CH) (36, 45). In contrast, the Nutritional Reference Value (NRV) specified in the EU food labeling regulations for VitD is 5 μg. However, we should note that EFSA sets DRV for the VitD as adequate intake, so that 97.5% of population avoid deficiency (46), but not insufficiency.

In the light of the alarming prevalence of regional VitD deficiency (1, 40, 44), we designed a study with aim to examine the factors affecting VitD status in the Caucasian population in winter and summer, focusing on objectively measured skin parameters and the consideration of a wide range of personal and lifestyle indicators. The results of present study will be implemented into new policies for public health interventions.

## 2. Materials and methods

### 2.1. Study design and timing

This prospective longitudinal study was carried out within the scope of research project Nutri-D: “Challenges in achieving adequate Vitamin D status in the adult population” (L7-1849) conducted with two observation periods, one in summer (September) and one in winter (January). The study protocol was compliant with the principles of the Declaration of Helsinki and was approved by the Ethics Committee of the Faculty of Applied Sciences (Approval No. 2018/4-ET-SK; ClinicalTrials.gov entry NCT03818594). The study was conducted for two consecutive years (2019–2020), with approximately half of the participants recruited in each year. Prior to enrolment, the participants were screened for inclusion/exclusion criteria. At the first visit (January), the participants signed the Informed Consent Form (ICF), and a fasting venous blood sample was collected for the determination of serum 25(OH)D concentration. At the second visit (September), blood sampling was repeated, skin parameters were measured, and the sqFFQ/SI and behavior questionnaire were completed.

It should be noted that the running of the study in 2020 coincided with the onset of the COVID-19 pandemic. Winter recruitment in 2020 was conducted before the virus outbreak in Slovenia, which led to an initial lockdown (March 12th until May 15th, 2020), and summer sampling occurred just before the second wave of infection, which led to a second lockdown in the autumn of 2020. Some pandemic measures were also in force during the summer months, which affected people’s lifestyles and holidays.

### 2.2. Study population

The invitation to participate in the study was posted on social media and on the website of the Nutrition Institute (Slovenia). The inclusion criteria were Caucasian race (Fitzpatrick skin type I–IV); age over 18 years; willingness to avoid artificial UVB sources; and willingness to follow all study procedures. The exclusion criteria were pregnancy or breastfeeding; marked sun avoidance (e.g., sun allergy); use of sunbed; intake of supplements or medications containing VitD, fish oil, or omega-3 fatty acids in the 3 months preceding study enrolment; regular (daily) consumption of VitD-enriched foods (fortified margarine or plant-based alternative milk); diet prescribed by dietitian/medical staff; adherence to special diets (veganism, a low-carbohydrate, high-fat diet (LCHF), and a caloric restriction diet; note: vegetarians were not excluded); current diseases of the kidneys, thyroid, digestive tract, osteoporosis and other bone diseases, and skin diseases and other diseases and conditions that affect the absorption and synthesis of VitD, and additional exclusion criteria for the winter time participation was vacation in (sub)tropical destination.

Of 338 subjects enrolled, 292 completed all the commitments and measurements in both seasons (33 could/would not participate in the second season, 3 dropped out for medical reasons, 3 because of pregnancy, and 7 were excluded because of supplement use). The blood collection and measurements of the study were organized in Ljubljana, so most of the participants (73.3%) were from the region of Central Slovenia.

### 2.3. Demographic questionnaire and food frequency questionnaire for the estimation of vitamin D intake

The survey included information on eating habits, food allergies, sociodemographic and socioeconomic status, self-reported body height and weight, and health status. Body height and weight were used to calculate the body mass index (BMI); subjects were categorized as: normal and underweight (< 24.9 kg/m^2^), overweight (25.0–29.9 kg/m^2^), and obese (> 30.0 kg/m^2^) (47). Physical activity levels were assessed using the short version of the International Physical Activity Questionnaire (IPAQ) (Slovenian translation in (48). As suggested by Craig et al. (49) physical activity levels were categorized as low, moderate, and high. The Fitzpatrick skin type questionnaire was used to assess self-reported skin color and sun reactivity (50).

Vitamin D intake was estimated using the validated Semi-Quantitative Food Frequency Questionnaire (sqFFQ/SI). The sqFFQ/SI questionnaire was designed to measure only VitD intake with capturing main contributors of VitD to the Slovenian diet and was validated against 5-day dietary record (51). Intake was analyzed dichotomously as below and above 5 μg per day.

### 2.4. Serum 25(OH)D concentration

A venous blood sample was collected after an overnight fast. Blood collection and analyses were carried out at an accredited medical diagnostic laboratory Adrialab/Synlab (Ljubljana, Slovenia), using a standard chemiluminescence method, the Architect 25-OH vitamin D chemiluminescent microparticle immunoassay (Abbott Ireland, Longford, Ireland), to determine the quantity of 25(OH)D in human serum. As provided in the technical documentation of the used assay kit, the correlation coefficient with the ID-LC–MS/MS method within the measurement interval of the assay (12–378 nmol/L) was *r* = 0.99 (95% CI: 0.99, 1.05). Vitamin D status was determined considering the 25(OH)D serum concentration according to the literature (33–35): deficient below 30 nmol/L (12 ng/mL); insufficient 30–50 nmol/L (12–20 ng/mL); sufficient 50–75 nmol/L (20–30 ng/mL); and optimal concentration above 75 nmol/L (30 ng/mL). The difference between the 25(OH)D concentration in January and September was divided into three quartiles (corresponding to the 25th and 75th percentiles).

### 2.5. Skin measurements

Measurements of skin color and of the level of melanin were made according to standard procedures using the DSM III Skin Colormeter (Cortex Technology ApS, Denmark) (52). Skin color was measured in the CIE L*a* b* color system, using three coordinates representing lightness (L) – values range from 0 (black) to 100 (white); the green-red axis (a) – positive values indicate the amount of red and negative values indicate the amount of green; and the blue-yellow axis (b) – yellow is positive and blue is negative (53). Melanin content was measured as the melanin index (MI) between 0 and 100; the higher the index value, the darker and more pigmented the skin.

The measurements were taken on the inner (sun-unexposed area) and lower outer (sun-exposed area) sides of the upper right arm. To comply with guidelines for measuring skin color (54), which suggest taking at least 3 measurements at each test site to account for variability within the body area, four measurements were taken at different locations of each test site, avoiding moles and discoloration, and their mean was included in the statistical analysis. The measurements on the exterior side of the arm were reflective of skin color altered by ultraviolet radiation (facultative pigmentation), whereas measurements on the interior side of the upper arm indicated innate skin color (constitutive pigmentation). Inner side of upper arm is commonly used for determination of constitutive skin color and it was shown that there is good intra-individual MI correlation with MI of other body parts (55). The device was calibrated before use using a white calibration plate provided by the manufacturer. The subjects were asked not to apply cosmetic products to the upper arm or to wear clothing with tight sleeves on the day of the measurements.

For the objective classification of skin color type, the individual typology angle (ITA) was calculated from the measurement of constitutional color in the interior upper arm area according to the following formula: ITA° = [arctan(L*-50)/b*] x 180/π. Caucasian skin color types can be classified into four groups, ranging from very light to tanned skin: very light >55° > light >41° > intermediate >28° > tanned skin >10° (56, 57). This classification is an objective and quantitative alternative to the more subjective Fitzpatrick skin phototype classification (58).

To assess the degree of sun exposure of individuals more quantitatively, the difference between the facultative and constitutional pigmentation was calculated as the difference between the melanin index (MI) at the sun-exposed (exterior) and unexposed (interior) areas of the upper arm: ΔMI = MI (exterior area of the upper arm) – MI (interior area of the upper arm).

### 2.6. Behavior questionnaire on sun exposure

The behavior questionnaire on sun exposure included the following questions: holiday country and exact dates of holidays in the last 2 months (‘holidays’ definition: more than 4 consecutive days of vacation time), categorized as: up to 6 days, 6–14 days, more than 14 days; hours of exposure to strong sun (in hours from 10 am to 4 pm) – separately for weekdays, weekend days, and holidays; wearing long sleeves as a protective measure against the sun (never, sometimes, always); and use of sun protection factor (SPF) products, level of SPF protection (below or above 30 SPF), and how often they use SPF products (never, sometimes, always). The questions about use of SPF were merged and categorized into: at least one response ‘never’; < 30 SPF and ‘sometimes’; > 30 SPF and ‘always’.

### 2.7. Statistical analyses

Data analyses were performed using IBM SPSS Version 27 (IBM SPSS, IBM Corp., Armonk, NY) (59). Categorical variables were expressed as frequencies and percentages [*N* (%)]. Binary logistic regression based on *a priori* knowledge was performed to analyse potential factors associated with the prevalence of low VitD status. The crude and modeled binary logistic regression method was used and odds ratios (OR) (with 95% confidence intervals) were calculated for the prevalence of serum 25(OH)D concentrations below 50 nmol/L and 75 nmol/L for winter and summer measurements, respectively. Winter (January) variables included sex, year of inclusion, BMI, individual typology angle category (ITA) inner upper arm, VitD intake, seasonal 25(OH)D concentration difference, and IPAQ. The winter regression model (Model W) included all the aforementioned factors, except IPAQ. The Summer (September) logistic regression was done using two models. The variables included in the crude calculations were same as for winter, with added variables related to behavior in the sun and skin pigmentation: number of holiday days, SPF and frequency of SPF use, and wearing long sleeves; and two cofactors: the difference between the melanin index (MI) on the outer and inner upper arm, and hours in full sun. In the summer regression Model S1, we included the same variables as in Model W, and quantitative measures of the difference in the MI upper arm (ΔMI), whereas in summer regression Model S2, we included same variables as in Model W, together with self-reported factors (number of holiday days, SPF and frequency of SPF use, wearing long sleeves, and hours in full sun). As in the winter model, IPAQ was not included in either Model S1 or Model S2. The change in serum 25(OH)D concentration between winter and summer was examined using repeated-measures ANCOVA and two models (C1&2), with winter serum 25(OH)D concentration as covariate. We used the same variables as in the summer regression models for suboptimal VitD status. Considering that ITA is related to the skin’s ability to pigment after UV exposure (60), the interaction between ITA and ΔMI was used in the modeling (model C1). In all comparisons, the significance was considered at *p* < 0.05.

## 3. Results

A total of 292 participants completed the study, of whom 38% were male and 62% were female. Enrolment took place in 2019 (70.2%) and 2020 (29.8%). The mean age (standard deviation) of participants at enrolment was 38.2 (±11.8) years, with a median of 37 years. Overall, 33.6% of the participants were overweight or obese, and the mean BMI was 24.1 (±SD 3.3). Insufficient or deficient wintertime VitD status (January) was found in 63.4% of the participants, with mean 25(OH)D concentration 44.5 nmol/L (±SD 17.2). In the summertime 50% of participants had below sufficient status, and the mean 25(OH)D concentration was 80.0 nmol/L (±SD 24.8). The mean (±SD) dietary intake of VitD was 2.9 ± 1.7 μg (median 2.5 μg), with the maximum intake of 12.72 μg per day. Other characteristics of the population are shown in Tables 1, 2.

**Table 1:** Descriptive characteristics of the study population (*N* = 292; Slovenia).

Parameter	Group	*N* (%)
Sex	Male	111 (38)
	Female	181 (62)
Year of inclusion	2019	205 (70.2)
	2020	87 (29.8)
Body mass index category	Normal and underweight (< 24.9 kg/m^2^)	194 (66.4)
	Overweight (25.0–29.9 kg/m^2^)	72 (24.7)
	Obese (> 30.0 kg/m^2^)	26 (8.9)
Education level	Primary or vocational school education	6 (2.0)
	Secondary education	52 (17.8)
	Vocational college	54 (18.5)
	University degree	127 (43.5)
	PhD, master’s, or specialization	53 (18.2)
Winter vitamin D status January	Deficiency (< 30 nmol/L)	61 (20.9)
	Insufficiency (30–50 nmol/L)	124 (42.5)
	Sufficiency (50–75 nmol/L)	93 (31.8)
	Optimal (> 75 nmol/L)	14 (4.8)
Summer vitamin D status September	Deficiency (< 30 nmol/L)	2 (0.7)
	Insufficiency (30–50 nmol/L)	14 (4.8)
	Sufficiency (50–75 nmol/L)	130 (44.5)
	Optimal (> 75 nmol/L)	146 (50.0)
Constitutive skin color, ITA (individual typology angle)	Very light (> 55°)	28 (9.6)
	Light (> 41°)	124 (42.5)
	Intermediate (> 28°)	71 (24.3)
	Tan (> 10°)	69 (23.6)

**Table 2:** Key descriptive characteristics of study participants expressed as continuous variables.

Variable	Mean (± standard deviation)
Body mass index	24.1 ± 3.8
Vitamin D intake/day (μg)	2.9 ± 1.7
Vitamin D concentration January (nmol/L)	44.5 ± 17.2
Vitamin D concentration September (nmol/L)	80.0 ± 24.8
Constitutive skin color, ITA	38.4 ± 15.7

ITA, individual typology angle measured on inner upper arm.

Factors affecting VitD status in the winter and summer seasons were examined using binary regression analysis. We calculated the seasonal OR, OR using a winter model (Model W), and two summer models (Models S1 and S2). In the summer Model S1, we added objectively quantifiable measurements of the difference in the melanin index between constitutive and facultative skin pigmentation as an indicator of sun exposure level. In the summer Model S2, we added self-reported variables: number of holidays, how often and what SPF factor they use, hours spent in full sun, and whether they wear long sleeves as a protective measure against the sun.

Significant differences in crude ORs for insufficient VitD status in winter were found for the following variables: BMI (higher in obese individuals); seasonal differences in 25(OH)D concentrations (higher in individuals with greater differences); and ITA (lower in tan and intermediate subjects) (Table 3). Daily VitD intakes greater than 5 μg showed a nonsignificant trend (*p* < 0.1) of lower OR intake of insufficiency. Model W included all the variables used in the crude calculation except IPAQ and showed similar OR to the crude calculations. The highest ORs was found for BMI and the lowest for ITA°. Obese participants were 7.92 (95% CI: 2.11, 29.67) times more likely to be VitD insufficient than normal or underweight participants, while individuals in the ‘intermediate’ and ‘tan’ ITA categories were significantly less likely to have VitD insufficiency than individuals in the ‘light’ ITA category (which is the most common in Slovenia).

**Table 3:** Prevalence of insufficient/sub-optimal vitamin D status in winter/summer season with odds ratios (OR) and 95% confidence intervals (CI) for crude variables and winter (Model W), and two summer models (Model S1 and S2).

	Winter insufficient vitamin D status (serum 25(OH)D concentration < 50 nmol/L)			Summer suboptimal vitamin D status (serum 25(OH)D concentration < 75 nmol/L)			
	Prevalence	Crude	Model W	Prevalence	Crude	Model S1	Model S2
	*N* (%)	OR (95% CI)	OR (95% CI)	*N* (%)	OR (95% CI)	OR (95% CI)	OR (95% CI)
Sex							
- Male	73 (65.8)	Ref	Ref	46 (41.4)	Ref	Ref	Ref
- Female	112 (61.9)	0.85 (0.52, 1.38)	1.14 (0.64, 2.05)	100 (55.3)	1.74* (1.08, 2.81)	0.88 (0.47, 1.66)	1.05 (0.55, 2.00)
Observation year							
- 2019	130 (63.4)	Ref	Ref	93 (45.4)	Ref	Ref	Ref
- 2020	55 (63.2)	0.99 (0.59, 1.67)	1.10 (0.60, 2.00)	53 (60.9)	1.88* (1.13, 3.13)	2.03* (1.05, 3.92)	1.77^#^ (0.91, 3.46)
Body mass index							
- Normal and underweight	112 (57.7)	Ref*	Ref*	89 (45.9)	Ref*	Ref*	Ref*
- Overweight	50 (69.4)	1.66^#^ (0.93, 2.96)	1.73^#^ (0.91, 3.28)	37 (51.4)	1.25 (0.73, 2.14)	1.68 (0.86, 3.25)	1.99* (1.01, 3.98)
- Obese	23 (88.5)	5.61* (1.63, 19.33)	7.92* (2.11, 29.67)	20 (76.9)	3.93* (1.51, 10.22)	4.4* (1.46, 13.28)	5.53* (1.77, 17.27)
Constitutive skin color, ITA							
- Very light	20 (71.4)	0.96 (0.41, 2.5)	0.82 (0.30, 2.2)	15 (53.6)	0.64 (0.28, 1.5)	0.40^#^ (0.15, 1.1)	0.34^#^ (0.12, 1.00)
- Light	88 (71.0)	Ref*	Ref*	80 (64.5)	Ref*	Ref*	Ref*
- Intermediate	40 (56.3)	0.53* (0.29, 0.97)	0.49* (0.25, 0.95)	32 (45.1)	0.45* (0.25, 0.81)	0.52^#^ (0.26, 1.0)	0.54^#^ (0.26, 1.11)
- Tan	37 (53.6)	0.47* (0.26, 0.87)	0.28** (0.14, 0.57)	19 (27.5)	0.21** (0.11, 0.40)	0.29* (0.14, 0.63)	0.32* (0.15, 0.70)
Vitamin D intake/day							
- Below 5 μg	172 (65.2)	Ref	Ref	137 (51.9)	Ref	Ref	Ref
- Above 5 μg	13 (46.4)	0.46^#^ (0.21, 1.02)	0.51 (0.21, 1.20)	9 (32.1)	0.44^#^ (0.19, 1.01)	0.31* (0.11, 0.86)	0.28* (0.10, 0.80)
Δconc. 25(OH)D							
- 1. Quartile (< 22.3 nmol/L)	33 (44.0)	Ref**	Ref**	58 (77.3)	Ref**	Ref**	Ref**
- 2. and 3. Quartile (22.3–47.0 mol/L)	99 (68.3)	2.74** (1.54, 4.87)	3.20** (1.69, 6.04)	78 (53.8)	0.34** (0.18, 0.64)	0.33* (0.17, 0.67)	0.30** (0.14, 0.62)
- 4. Quartile (>47.0 nmol/L)	53 (73.6)	3.55** (1.77, 7.11)	6.78** (2.98, 15.44)	10 (13.9)	0.05** (0.02, 0.11)	0.05** (0.02, 0.13)	0.06** (0.02, 0.15)
IPAQ			NIM			NIM	NIM
- Low	21 (70.0)	Ref		18 (60.0)	Ref		
- Moderate	77 (67.5)	0.89 (0.37, 2.14)		62 (54.4)	0.79 (0.35, 1.8)		
- High	87 (58.8)	0.61 (0.26, 1.43)		66 (44.6)	0.54 (0.24, 1.19)		
Number of Holiday Days						NIM	
- Up to 6 days	69 (65.7)			64 (61.0)	Ref*		Ref
- 6–14 days	59 (64.1)			45 (48.9)	0.61^#^ (0.35, 1.08)		0.88 (0.43, 1.81)
- More than 14 days	57 (60.0)			37 (39.0)	0.41* (0.23, 0.72)		0.85 (0.41, 1.75)
SPF and frequency of SPF use						NIM	
- At least one answer ‘never’	45 (56.3)			35 (43.8)	Ref^#^		Ref
- < 30 SPF and ‘Sometimes’	124 (66)			94 (50.0)	1.29 (0.76, 2.18)		0.91 (0.47, 1.78)
- > 30 SPF and ‘always’	16 (66.7)			17 (70.8)	3.12* (1.17, 8.36)		2.34 (0.63, 8.65)
Wearing long sleeves						NIM	
- Never	131 (62.7)			101 (48.3)	Ref		Ref*
- Sometimes	41 (60.3)			34 (50.0)	1.07 (0.62, 1.85)		1.47 (0.74, 2.92)
- Always	13 (86.7)			11 (73.3)	2.94^#^ (0.91, 9.53)		6.15* (1.36, 27.84)
Δ Melanin index					0.9* (0.84, 0.96)	0.89* (0.82, 0.97)	NIM
Hours in full sun					0.69** (0.57, 0.84)	NIM	0.75* (0.59, 0.96)

Ref, reference value (odds ratio = 1); NIM, not in the model; Δconc, seasonal difference in the concentration of serum 25(OH)D; 25(OH)D, 25 hydroxy vitamin D; SPF, sun protection factor; ITA, individual typology angle measured on inner upper arm; Δ Melanin Index (ΔMI), difference in Melanin Index between sun-exposed and sun-unexposed side of arm; OR, odds ratio; CI, confidence interval; VitD, vitamin D; IPAQ, international physical activity questionnaire; *p* values, ** < 0.001, * < 0.05, ^#^ < 0.1. In addition to winter Model W, summer Model S1 included objective measures for sun exposure while Model S2 included subjective measures for sun exposure. Model W included variables: sex, observation year, body mass index, vitamin D intake/day, Individual typology angle, difference between summer and winter 25(OH)D concentration. Model S1 included variables: sex, observation year, body mass index, vitamin D intake/day, individual typology angle, difference between summer and winter 25(OH)D concentration, and Difference in Melanin index. Model S2 included variables: sex, observation year, body mass index, vitamin D intake/day, Individual typology angle, difference between summer and winter 25(OH)D concentration., number of holiday days, SPF and frequency of SPF use, wearing long sleeves, and hours in full sun.

For summer, in addition to the crude analysis, two models were configured, one based on measured variables (Model S1), and another based on self-reported variables (Model S2). Sex was significant only in the crude analyses, but not when adjusted with other factors (Models S1 and S2) (Table 3). On the contrary, daily VitD intake was not significant as crude value, but adjusted with other factors in Model S1 and S2, became significant (*p* < 0.05). In both summer models, the year of observation influenced VitD status. The participants observed in 2020, which included the first summer of the Covid-19 pandemic, were approximately twice as likely to have sub-optimal VitD status. In Model S1, the difference in melanin index (MI) between the inner and outer upper arm was a significant variable (analyzed as a continuous variable); at the same time ITA remained significant in individuals with ‘tan’ skin [0.29 (95% CI: 0.14, 0.63)], when compared to reference ‘light’ skin. The odds ratio for BMI remained highest for the obese subjects at OR 4.4 (95% CI: 1.46, 13.28). The second summer model (Model S2) included variables that were significant in the crude analyses. The analyses showed that the odds ratios were consistent with Model S1 for BMI (OR 5.33; 95% CI: 1.71, 16.59) and the ITA for ‘tan’ (OR 0.33; 95% CI: 0.15, 0.73). In this model, the largest odds ratio for deficiency of VitD was attributed to wearing long sleeves as a protective measure against the sun. Those who reported wearing long sleeves were 5.98 (95% CI: 1.33, 27.03) times more likely to have suboptimal VitD status. The number of hours spent in full sun was also significant.

**Figure 1:**
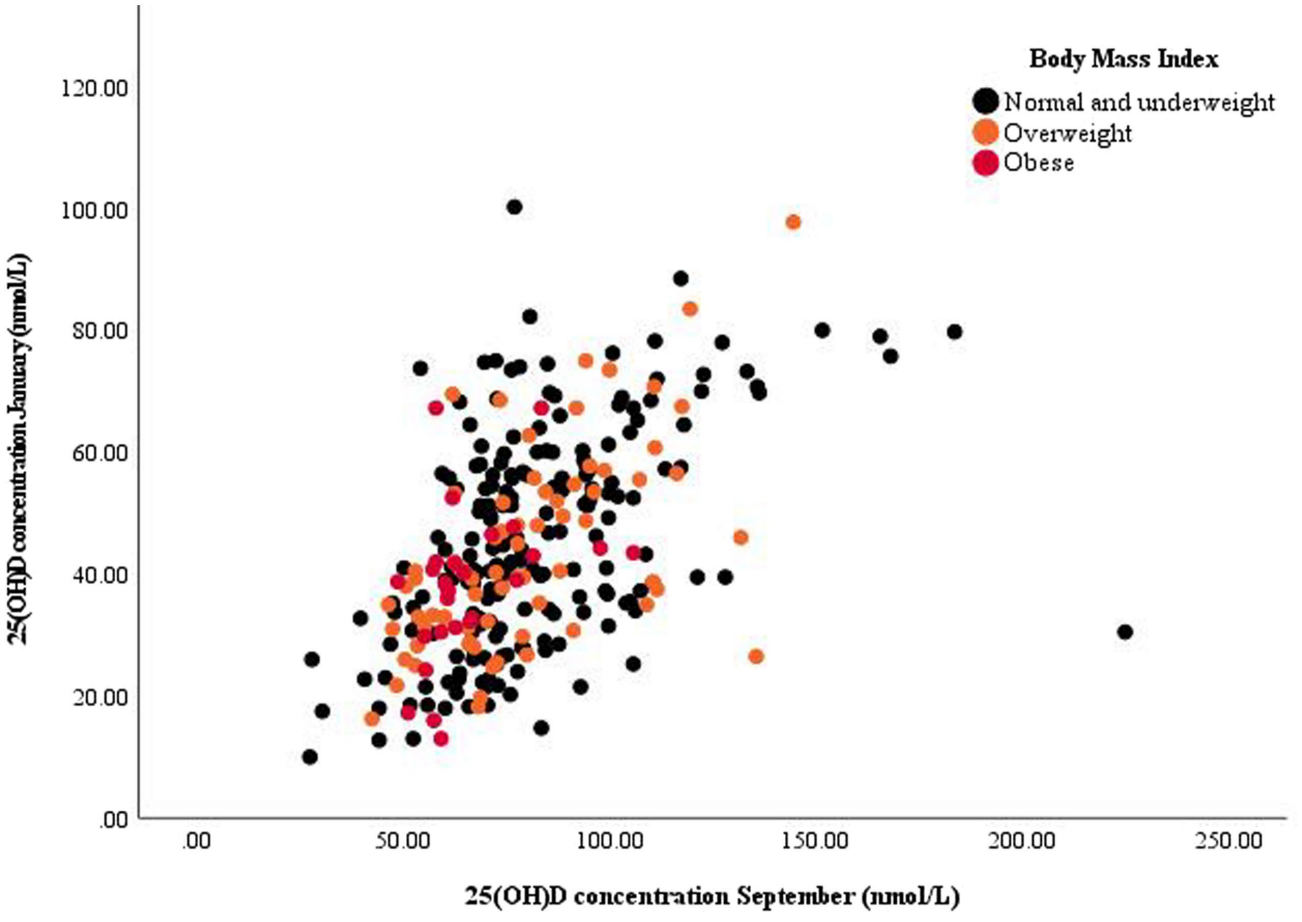
Scatter plot of summer and winter serum 25-hydroxy vitamin D [25(OH)D] concentration with annotated datapoints according to body mass index categories.

We also examined parameters related to interindividual differences in serum 25(OH)D concentration between winter and summer. Figure 1 presents a plot of summer and winter serum 25(OH)D concentration, while the results of the repeated-measures ANCOVA analyses are shown in Table 4. Statistically significant predictors in the objective model C1 were BMI, year of observation, and ITA*ΔMI, while sex was non-significant with *p* = 0.057. In the subjective model C2, predictors of seasonal difference in serum 25(OH)D concentration were BMI, sex, ITA, and number of holiday days.

**Table 4:** Predictors of change in serum 25-hydroxy vitamin D [25(OH)D] concentration between winter and summer for regression models C1 and C2.

	Model C1		Model C2	
	*F*	*P*	*F*	*P*
Intercept	92.0	< 0.001	73.5	< 0.001
Sex	3.66	0.057	5.15	0.024*
Observation year	4.98	0.026*	1.87	0.17
Body mass index	4.49	0.012*	4.66	0.010*
Vitamin D intake/day	1.30	0.26	1.74	0.19
Constitutive skin color, ITA	4.60	0.001*	3.60	0.014*
Δ Melanin index				
Number of holiday days			9.81	< 0.001*
SPF and frequency of SPF use			1.97	0.14
Wearing long sleeves			0.97	0.38
Hours in full sun			3.12	0.078

Results of repeated-measures ANCOVA using winter serum 25(OH)D concentration as covariate. SPF, sun protection factor; ITA, individual typology angle measured on inner upper arm; Δ Melanin Index (ΔMI): difference in Melanin index between sun-exposed and sun-unexposed side of arm; *significant at *p* < 0.05. Model C1 included variables: sex, observation year, body mass index, vitamin D intake/day, and individual typology angle (ITA) * Δ Melanin Index (as composite variable). Model C2 included variables: sex, observation year, body mass index, vitamin D intake/day, individual typology angle, number of holiday days, SPF and frequency of SPF use, wearing long sleeves, and hours in full sun.

## 4. Discussion

Public health interest in vitamin D is increasing due to its versatile body functions and the high prevalence of deficiency worldwide (3, 7). For example, a nationally representative Slovenian Nutrihealth study showed that approximately 80% of adults have insufficient VitD status in winter and 60% have suboptimal VitD status in summer (1).

While several studies have addressed VitD status in population-based cross-sectional studies [see a very recent review by Cashman on this topic (61)], few studies have followed seasonal changes in serum 25(OH)D concentration with a prospective cohort study design, and have generally focused on specific population subgroups or had limited sample sizes. The largest prospective cohort study was conducted by MacDonald et al., (62) who studied 314 Caucasian postmenopausal women and emphasized the importance of BMI. Andersen et al. (63) examined seasonal changes in VitD status in 54 Danish adolescent girls and 52 elderly women, focusing on both sun habits and VitD supplementation. Pittaway et al. (64) studied 91 older subjects (71% women, aged 60–85 years) in Tasmania and also noted the importance of VitD supplementation; supplement use was associated with mostly smaller seasonal variations in serum 25(OH)D concentration. Brustad et al. (65) followed VitD status in 60 Norwegian adults (73% women, age 20–60 years) with bi-monthly observation time and reported that high dietary VitD intake largely masks seasonal variations. On the other hand, Wolman et al. (66) included only subjects who supplemented VitD, but their study was very specifically focused on elite ballet dancers (n = 19; 65% women); the authors reported notable seasonal differences in VitD status, but did not focus on skin parameters. Our literature search did not identify any prospective cohort studies addressing seasonal changes in VitD status in the general adult population and using objective methods to measure skin color and pigmentation. VitD supplementation is a known major parameter linked to serum 25(OH)D concentration (67), therefore we wanted to focus on other parameters related to VitD status, and to exclude the effects of supplementation by study design.

The current study therefore used a prospective cohort design with measurements during the winter and summer seasons in subjects who did not supplement with VitD. Although VitD also occurs naturally in foods, the average daily dietary intake of this vitamin in Slovenian adults is only 2.9 μg (40) – much lower than typically supplemented dosages (25 μg) (68, 69). Nevertheless, we also followed the dietary intake of VitD with foods using a validated food frequency questionnaire (51). Serum 25(OH)D concentration was measured as a marker of VitD status, as were other characteristics, such as skin pigmentation and sun behavior. The strength of the study also lies in the use of objective measures of skin color and pigmentation. Using ITA, we were able to account for different levels of constitutive skin color in the Caucasian population. Given the study objectives, we recruited subjects with a wide variability in the parameters studied, and therefore did not use a nationally representative sampling approach. Overall, 62% of the study participants were women. BMI was self-reported, and 66% of participants fell into the normal/underweight category, 25% into the overweight category, and 6% into the obese category, the mean BMI of the entire population was 24.1 (SD ± 3.8).

Although the observed prevalence of inadequate VitD status should not be generalized to the population, it is interesting to compare the observed prevalence with the nationally representative Nutrihealth (NH) study (1). The mean 25(OH)D concentration in January was 44.5 nmol/ L comparing to 36.7 nmol/L in NM study (winter time), for the September, the mean concentration was 80.0 nmol/L, comparing to 70.4 n mol/L in NH study (summer time)The prevalence of suboptimal VitD status (25(OH)D concentration < 75 nmol/L) in our study was 95.2% in winter (NH: 98.0%) and 50.0% in summer (NH: 62.6%). More notable differences were observed in the prevalence of insufficient VitD status (<50 nmol/L), which was 63.4% in winter (NH: 81.6%) and 5.5% in summer (NH: 25.3%). These differences can be explained not only by the recruitment approach and the study population, but also by the observation periods. While the Nutrihealth study recruited subjects over the entire calendar year, our study focused on two narrow sampling periods – January for the winter season and September for the summer season. This approach was chosen to minimize within-season variability in environmental factors.

Regression analyses modeling was used to identify the most important factors affecting seasonal VitD status in our population group. Large differences in winter and summer VitD status, therefore the analyses were made with different cutoff targets for each season. The cutoff value was set at <50 nmol/L (insufficient status) for winter, and at <75 nmol/L (suboptimal status) for summer, because of the seasonal differences in environmental factors and human behavior, the regression models were constructed as season specific. We used one winter model (Model W), which did not include variables related to sun exposure/protective behavior, and two summer models (Model S1 and S2): the first considering only objectively measured variables, and the second taking into consideration subjectively measured parameters. All the models included the usual variables that may influence VitD status, such as sex, BMI, VitD intake, and skin color category (ITA), along with seasonal differences in 25(OH)D concentration. Considering the differences in environmental and lifestyle factors, the year of observation was also included in all the models. In this regard, it should be noted that the second year of the study coincided with the Covid-19 pandemic and its associated restrictions, which affected the behavior of the subjects during the summer sampling period. Given the prospective study, we also evaluated seasonal differences in serum 25(OH)D concentration, using repeated measures analyses.

Our results show that BMI is a strong predictor of VitD status in both winter and summer periods. Compared with normal/underweight individuals, obese individuals were several times more likely to have insufficient VitD status in winter (model W; OR 7.9), and suboptimal VitD status in summer (Models S1 and S2; OR 4.4 and 5.5, respectively; Table 3). BMI was also confirmed as a significant variable in repeated measures analyses of seasonal changes in serum 25(OH)D concentration (Table 4). BMI has previously been described as a predictor of VitD status (25, 26, 28, 62, 67, 70–72) and of seasonal differences in serum 25(OH)D concentration (62). Several factors could contribute to the lower 25(OH)D concentration in individuals with a higher BMI, including a larger pool for the accumulation of VitD in adipose tissues, and lifestyle covariates (i.e., less outdoor activity due to lower mobility), but the association remains controversial (71, 73). In conclusion, BMI is a strong predictor, but we should interpret results with some caution since BMI was self-reported variable.

In our study, sex was not found to be a significant predictor of VitD status either in winter or in summer (after adjustment for other factors; Table 3), but there was an association with seasonal changes in serum 25(OH)D concentration (Table 4). We could find no other literature with prospective cohort data on this topic, and data from cross-sectional studies vary widely. In some studies, although sex was reported as a significant predictor of VitD status, the direction of the effect may be in the opposite direction (61). However, in the NH study (1) and some other studies (25, 26), lower VitD status was found in women.

Daily dietary intake of VitD (with food) was low (median 2.5 μg) and comparable to epidemiological data for Slovenian adults (NH: 2.7 μg) (40, 44). Also, the maximum intake was 12.72 μg per day, which is well below the recommended intake of 20 μg per day (45). Nevertheless, dietary VitD intake showed tendency (*p* = 0.057) to be significantly correlated with VitD status as a sole variable. Surprisingly in the summer models, the higher dietary VitD intake was correlated with optimal VitD status (Models S1 and S2). This was unexpected, since usually VitD intake is bigger predictor of VitD status in winter, in the absence of endogenous VitD synthesis which is the major contributor to VitD source (14–16). Literature shows, that supplementation with higher doses of VitD is a predictor of personal VitD status, whereas the effect of lower dietary VitD intake with foods is not consistent across studies (63, 74).

In our study, we used ITA as an objective and quantitative method to classify constitutional skin pigmentation. It has six classes ranging from ‘very light’ to ‘dark’ (56), but because the study was conducted in Slovenia with a Caucasian population, our sample did not include the ‘brown’ and ‘dark’ categories. ITA is used as an objective alternative to the subjective Fitzpatrick skin phototype (58), which is based on self-reported pigmentation and solar reactivity of the skin (50), and has been used as a measure of skin pigmentation in several studies (75). It should be noted that the ITA and the Fitzpatrick phototype (FT) classifications are not completely equivalent; whereas the FT is easier to use in a clinical setting, ITA is preferred for its objective classification of skin type in research (58, 76, 77). The analyses were performed using the base category of ‘light’, which was the most common in our study group. The odds ratios for lower VitD status decreased with darker skin pigment (lower ITA) in both winter and summer (Table 3). The lowest OR for insufficient VitD status (winter Model W) or suboptimal VitD status (summer Models S1 and S2) were observed in the ‘tan’ category: 0.28, 0.29, and 0.33 for Models W, S1, and S2, respectively. These observations add new and interesting findings to the existing knowledge. Previous studies that also considered skin color type compared individuals of different ethnicities and showed that individuals with more pigmented skin living in the same conditions had lower VitD status compared to Caucasians (24–28). To our knowledge, only one study has examined correlations with skin color in the Caucasian population: Dimakopoulos et al. (74) conducted a cross-sectional examination of factors affecting serum 25(OH)D concentrations and showed similar trends – the likelihood of lower VitD status was inversely related to darker skin. The observed results suggest that darker constitutive skin color in the Caucasian population is related to better VitD status, but there is likely a tipping point at which darker constitutive skin color no longer works in favor, but limits the production of 25(OH)D. It should also be noted that darker skin types tend to be less sun-reactive than lighter skin types, i.e., they have lower erythema sensitivity and better tanning ability; consequently, these individuals may be more susceptible to riskier sun habits and less likely to use sun protection measurements, (78) which could also contribute to lower odds for VitD deficiency.

In our study the melanin index was used as a measure of pigmentation; the difference between constitutive and facultative pigmentation was measured as ΔMI to objectively assess the degree of sun exposure of individuals and was included in the objective models S2 and C2. Dosimeters can be used to objectively quantify sun exposure, but their usage in larger and longer-term studies is challenging (63), whereas ΔMI can be easily measured during a single visit without burdening subjects on a daily basis during the research. The ΔMI was found to predict both seasonal change in serum 25(OH)D (Table 4; Model C2), and VitD status in summer (Table 3; Model S2).

To gain further insight, we also examined a set of self-reported variables related to peoples’ behavior and lifestyles (Models S1 and C1). In contrast to some other studies (25, 26, 70, 74), physical activity level was not found to be a significant predictor of VitD status, and therefore was not included in the modelling. On the other hand, the number of summer holiday days and the use of SPF were significant in the crude analyses of OR for summer low VitD status, but not when adjusted with other factors. We could hypothesize that this is also due to the interdependence of these two factors. On the other hand, the number of summer holiday days was strongly associated with individual seasonal changes in serum 25(OH)D concentration (Table 4; Model C2, *p* < 0.001). This was not the case with the use of SPF (*p* = 0.14). Some other studies also found no association between the use of SPF and VitD status (79), or sunscreen use was found to be a predictor of serum 25(OH)D, but normal sunscreen use was not associated with insufficient VitD status (80). Another subjectively reported parameter examined in our study was the hours of sun exposure between 10 am and 4 pm during summer, when the intensity of UV-B light is strong enough for VitD biosynthesis (81). This parameter was marginally significantly associated with seasonal changes in serum 25(OH)D concentration (Table 4; Model C2, *p* = 0.078), but significantly associated with summer VitD status (Table 3, Model S2, *p* < 0.05). Similar observations had previously been reported by Dimakopoulos et al. (74) This was also true for wearing long sleeves as a precautionary measure, which was identified as a very important parameter associated with VitD status in summer. Wearing long sleeves in summer increased the odds of suboptimal VitD status by 5.98-fold (95% CI: 1.33, 27.03) (Table 3, Model S2). Similarly, the observations of Datta et al. showed that daily self-reported summer clothing habits in summer, together with sun exposure, most influenced 25(OH)D concentration (27). Some other studies also associated body exposure to intense sunlight with VitD status (82, 83). It should be noted that caution should be exercised with increased sun exposure because of the risk of skin cancer, and that there is a fine line between beneficial UVB exposure and health risks, such as sunburn and skin cancer. It should also be noted that acute overexposure to sunbathing is less effective for VitD biosynthesis than more frequent, lower-level exposure (84), which is a viable option for safer sun exposure. However, debates on this topic are conflicting and indicate that there is no safe level of UVB exposure (85, 86).

We have mentioned several strengths of the study; in particular, the prospective cohort design, the control for VitD intake (exclusion of supplementation), and the use of objective measurements, but we should also point out some limitations of the study. Considering the population in our region, the study population consisted only of Caucasian adults, which means that we did not have access to subjects with dark brown and black skin color. On the other hand, previous studies have highlighted skin phototype as an important predictor of VitD status when comparing different ethnic populations (24–29), but this issue has not been adequately addressed within Caucasian population where differences in skin color are less pronounced. The data on body weight and height (used for calculation of BMI) was self-reported and not measured. We should also mention that VitD intake was not assessed from weighted food records, which are considered the gold standard method. VitD is mainly found in foods that are not consumed on a daily basis, which means that the collection of food records would be needed for several days, imposing a significant burden on the study participants. Therefore, dietary VitD intake was estimated using a validated semi-structured FFQ (51) which required only a single administration. In addition, the method used for objective assessment of summer sun exposure has some limitations. The difference in constitutive and facultative skin pigmentation has been used as an indicator of individual sun exposure, but the development of skin pigmentation varies between different skin color types and may reach a plateau above which an additional UV dose results in little or no additional pigmentation (75). This limitation could be overcome by using UV dosimeters (24, 63, 72), which was not possible in our study because of the large sample size. We should mention that dosimeters are usually used for rather short periods, whereas the half-life of serum 25(OH)D is measured in weeks (87). Long-term use of UV dosimeters would impose a significant burden on the participants, with high risks of noncompliance use during the most intense sun exposure (e.g., sunbathing).

## 5. Conclusion

VitD deficiency is highly prevalent around the world. Factors affecting seasonal VitD status have been studied in Caucasian adults. While previous studies have mostly used a cross-sectional design and subjective assessment of skin pigmentation, we conducted a longitudinal cohort study of subjects without VitD supplementation with summer and winter observation periods. Using a range of objective measures, we demonstrated that VitD status is hugely influenced by several personal parameters, limiting the usefulness of generalized population-level recommendations. While seasonal differences and a higher risk of VitD deficiency in winter are well known, we showed that skin pigmentation in both summer and winter is related to VitD status and to seasonal changes in serum 25(OH)D concentration. The general observation was that in the Caucasian population, darker constitutional skin color means lower odds of lower VitD status. Another key factor that should be given special consideration in future public health recommendations is body mass index. The results of the study will be used in the development of a screening tool to identify individuals at higher risk of insufficient VitD status. We used several objective methods to assess skin color and sun exposure behavior that will support further studies. Future research should primarily use objectively measured parameters and wider variability in skin color types.

## Data availability statement

The raw data supporting the conclusions of this article will be made available by the authors, without undue reservation.

## Ethics statement

The studies involving humans were approved by the Institutional Ethics Committee of VIST – Faculty of Applied Sciences (protocol code VITAD-01-2018, Approval No. 2018/4-ET-SK on 8th January 2019) and included in the ClinicalTrials.gov register under the entry NCT03818594. The studies were conducted in accordance with the local legislation and institutional requirements. The participants provided their written informed consent to participate in this study.

## Author contributions

KŽ and IP: conceptualisation. IP, KŽ, and MH: methodology. MH: validation and formal analysis. MH, TP, IP, and KŽ: investigation. IP and KŽ: resources. MH: data curation and writing—original draft preparation. IP, KŽ, and TP: writing—review and editing. MH: visualization. KŽ and IP: supervision. All the authors have read and agreed to the published version of the manuscript.
